# The old Iron Curtain split our continent in half: the new ones could tear apart our nations

**DOI:** 10.1093/eurpub/ckz237

**Published:** 2020-01-24

**Authors:** Rok Hrzic, Helmut Brand

**Affiliations:** 1 Department of International Health, Maastricht University, Care and Public Health Research Institute – CAPHRI, Maastricht, The Netherlands; 2 Prasanna School of Public Health, Manipal Academy of Higher Education, Manipal, Karnataka, India

In August 1989, the Iron Curtain began to crack. Thousands of East Germans were overstaying their summer vacations near Lake Balaton, resolved not to return. Fearing the coming winter and their resources already stretched thin by Romanian refugees, the Hungarian government had to act. The solution took the form of a ‘pan-European picnic’ near the Austrian–Hungarian border town of Sopron, which would temporarily suspend border controls and give the East Germans a chance to flee to the West.^1^ A German refugee crisis in the heart of Europe seems incredible today. But reconstructed mortality data shines a light on the dire state of East German health in the 1980s.^2^

During the first half of the post-war period, the East German healthcare system kept up with the west in preventing infant mortality and deaths due to infectious disease. Life expectancy in East Germany was as at least as high as in West Germany.^2^ However, after the epidemiological transition unfolded and the living standards improved, cardiovascular disease emerged as the leading cause of death throughout Europe. Western Europe, connected to the global markets and the worldwide medical community, would rapidly deploy advances like regular blood pressure check-ups, coronary surgery, beta blockers and thrombolytic drugs and improved emergency services. This reduced mortality for the middle-aged and the old and led to increased life expectancy. Meanwhile, life expectancy increases stalled in eastern Europe.^3^ By 1989, the stagnation resulted in an approximately 4-year gap in life expectancy between East and West German men.

The fall of the Iron Curtain resulted in the liberalization of governments and markets throughout Eastern Europe, and many of the newly independent countries joined the European Union (EU) after 2004. This coincided with increases in life expectancy in the region over the past three decades. In reunified Germany, former East Germany caught up with the west. In the post-2004 EU Member States, the rate of life expectancy increases matched the old Member States on average, but only rare exceptions followed East Germany in catching up.^4^ Why did not more new Member States see their life expectancy converge with the west? For one, we could argue that the example of German reunification is extreme. The reunification policies, especially welfare and infrastructure policy, led to an estimated transfer of 1 trillion EUR from western to eastern Länder. A transfer of resources on this scale from old to new Member States did not occur in the last 30 years and is after the experience of the Eurozone crisis politically unimaginable.

However, imagining this lack of convergence as a problem of exclusively new EU Member States is casting the issue too narrowly. It is not only places east of the old Iron Curtain that would benefit from knowing how to engineer a convergence in life expectancy. Regional life expectancy gaps *within* several old EU Member States today exceed that between East and West Germany before reunification. Regional life expectancy differences for men in 2017 exceeded 4 years in Belgium, Czechia and Spain, as well as Germany, which has the most experience closing, such gaps, and where a significant North-South gradient is now present.^4^ Beyond supporting action on the moral obligation to remove any unnecessary and unjust differences in population health, understanding drivers of convergence is important for other reasons too. Regional divides in life expectancy are likely associated with disparities in other measures of economic and social well-being. It is not unreasonable to expect that the same geographic disparities also feed into what some commentators describe as the growing political polarization throughout the EU over the past few years.^5^

What can be done? First, the concept of eastern and western Europe needs to be retired for good. New EU Member States may be saddled with a life expectancy debt as a consequence of their past stagnation but are today improving at least as fast as old EU Member States. Second, the focus on individual socioeconomic characteristics as determinants of health needs to be complemented with geographic perspectives that include the physical, social, economic and political contexts of individuals, which are just as influential in driving health outcomes. They deserve more research and policy attention. Finally, we need to acknowledge that the problem of convergence—either in life expectancy or other welfare measures—is a problem for all EU Member States to solve. It is a problem that is likely driven by broad political and economic structures that affect the health and well-being of places and individuals alike. We need to identify measures that can effectively support places and people falling behind, no matter what part of Europe they are in. Ignored, the current trends could lead to greater disparities, greater retrenchment and continual political crises.

*Conflicts of interest*: None declared.
